# The expression and regulation of matrix metalloproteinase-3 is critically modulated by *Porphyromonas gingivalis* lipopolysaccharide with heterogeneous lipid A structures in human gingival fibroblasts

**DOI:** 10.1186/1471-2180-13-73

**Published:** 2013-03-30

**Authors:** Thanuja D K Herath, Yu Wang, Chaminda J Seneviratne, Richard P Darveau, Cun-Yu Wang, Lijian Jin

**Affiliations:** 1 Faculty of Dentistry, Periodontology, The University of Hong Kong, 34 Hospital Road, Hong Kong SAR, China; 2 Department of Pharmacology and Pharmacy, Faculty of Medicine, The University of Hong Kong, Hong Kong SAR, China; 3 School of Dentistry, University of Washington, Seattle, WA, USA; 4 School of Dentistry, University of California, Los Angeles, CA, USA

**Keywords:** Periodontal disease, *P. gingivalis* LPS, Lipid A heterogeneity, MMPs, Human gingival fibroblasts

## Abstract

**Background:**

*Porphyromonas gingivalis* lipopolysaccharide (LPS) is a crucial virulence factor strongly associated with chronic periodontitis which is the primary cause of tooth loss in adults. It exhibits remarkable heterogeneity containing tetra-(LPS_1435/1449_) and penta-(LPS_1690_) acylated lipid A structures. Human gingival fibroblasts (HGFs) as the main resident cells of human gingiva play a key role in regulating matrix metalloproteinases (MMPs) and contribute to periodontal homeostasis. This study investigated the expression and regulation of MMPs1-3 and tissue inhibitors of MMP-1 (TIMP-1) in HGFs in response to *P. gingivalis* LPS_1435/1449_ and LPS_1690_ and hexa-acylated *E. coli* LPS as a reference. The expression of MMPs 1–3 and TIMP-1 was evaluated by real-time PCR and ELISA.

**Results:**

The MMP-3 mRNA and protein were highly upregulated in *P. gingivalis* LPS_1690_- and *E. coli* LPS-treated cells, whereas no induction was observed in *P. gingivalis* LPS_1435/1449_-treated cells. On the contrary, the expression of MMP-1 and −2 was not significantly affected by *P. gingivalis* LPS lipid A heterogeneity. The TIMP-1 mRNA was upregulated in *P. gingivalis* LPS_1435/1449_- and *E. coli* LPS-treated cells. Next, signal transduction pathways involved in *P. gingivalis* LPS-induced expression of MMP-3 were examined by blocking assays. Blockage of p38 MAPK and ERK significantly inhibited *P. gingivalis* LPS_1690_-induced MMP-3 expression in HGFs.

**Conclusion:**

The present findings suggest that the heterogeneous lipid A structures of *P. gingivalis* LPS differentially modulate the expression of MMP-3 in HGFs, which may play a role in periodontal pathogenesis.

## Background

Periodontal disease is a bacterially induced and highly common chronic inflammatory condition in humans, and severe periodontal disease (periodontitis) remains the major cause of tooth loss in adult population worldwide [1]. Dysregulated host response to pathogenic plaque biofilm critically contributes to destructive inflammation resulting in tissue damage and alveolar bone loss [2]. *Porphyromonas gingivalis* is a keystone periodontal pathogen in the mixed microbial community and it releases copious amount of lipopolysaccharide (LPS) which perpetually interacts with host cells, thereby significantly contributing to periodontal pathogenesis [1-4].

LPS is a potent immuno-inflammatory modulator which causes serious complications in host. It is comprised of three major components viz. outermost O-antigen, core oligosaccharide regions and innermost lipid A [3]. Lipid A is the biologically most active component of LPS that imparts the endotoxin activity. Its structure differs widely among Gram-negative bacteria species depending on the differences in composition of attached fatty acids, number of phosphorylation sites and substituted groups attached to the phosphate residues [3]. The canonical lipid A structure in *Escherichia coli* LPS is a hexa-acylated diphosphorylated glucosamine disaccharide. Previous studies have shown that *P. gingivalis* possesses highly heterogeneous lipid A structures containing penta-acylated LPS_1690_ and tetra-acylated LPS_1435/1449_, and this structural discrepancy may critically account for contrasting biological activities induced by *P. gingivalis* LPS [3,4].

Human gingival fibroblasts (HGFs) are the major cell type in human gingiva [5-7]. They play a key role in maintenance and remodeling of extra cellular matrix (ECM) by producing various structural components, such as collagen, elastin, glycoprotein and glycosaminoglycans. In addition, HGFs also synthesize and secrete various members of matrix metalloproteinases (MMPs) in response to *P. gingivalis* LPS challenge, which ultimately contribute to periodontal tissue destruction [8]. MMPs are a family of structurally and functionally related proteolytic enzymes containing a zinc-binding catalytic domain and they are active against the components of ECM [8-10]. The activity of MMPs is largely regulated by several naturally occurring inhibitors like tissue inhibitors of metalloproteinases (TIMPs) [11]. Overall, there is a remarkable balance between MMPs and TIMPs in periodontal connective tissues and disturbance of this balance is therefore critically implicated in the destruction of periodontal tissues [12,13]. In normal conditions, MMPs are involved in the remodeling and turnover of periodontal tissues under the strict control of TIMPs, which bind specifically to the active site of the enzyme thereby maintaining the equilibrium between degradation and regeneration of ECM [8,14]. Increased production of MMPs 1–3 is observed in chronic inflammatory condition such as periodontitis that results in excessive connective tissue breakdown [14,15]. MMPs such as MMP-1, -2, -3, -9 and −13 are synthesized in periodontal tissues in response to periodontopathic bacteria like *P. gingivalis.* Previous studies have suggested that LPS could regulate the MMP expression in various host cell types including HGFs [10,16].

Currently, there are no studies on the role of *P. gingivalis* LPS lipid A heterogeneity with respect to expression of MMPs in HGFs. The present study therefore aimed to investigate the expression and regulation of MMPs 1–3 and TIMP-1 in HGFs in response to the different isoforms of *P. gingivalis* LPS_1435/1449_ and *P. gingivalis* LPS_1690_ as well as *E. coli* LPS as a reference. This study sheds light on the regulation of MMP expression and underlying signal transduction pathways in HGFs in response to heterogeneous *P. gingivalis* LPS, which could have important implications in the pathogenesis of periodontal disease.

## Results

### Heterogeneous *P. gingivalis* LPS lipid A structures differentially modulate MMPs 1–3 and TIMP-1 mRNAs

**Figure 1:**
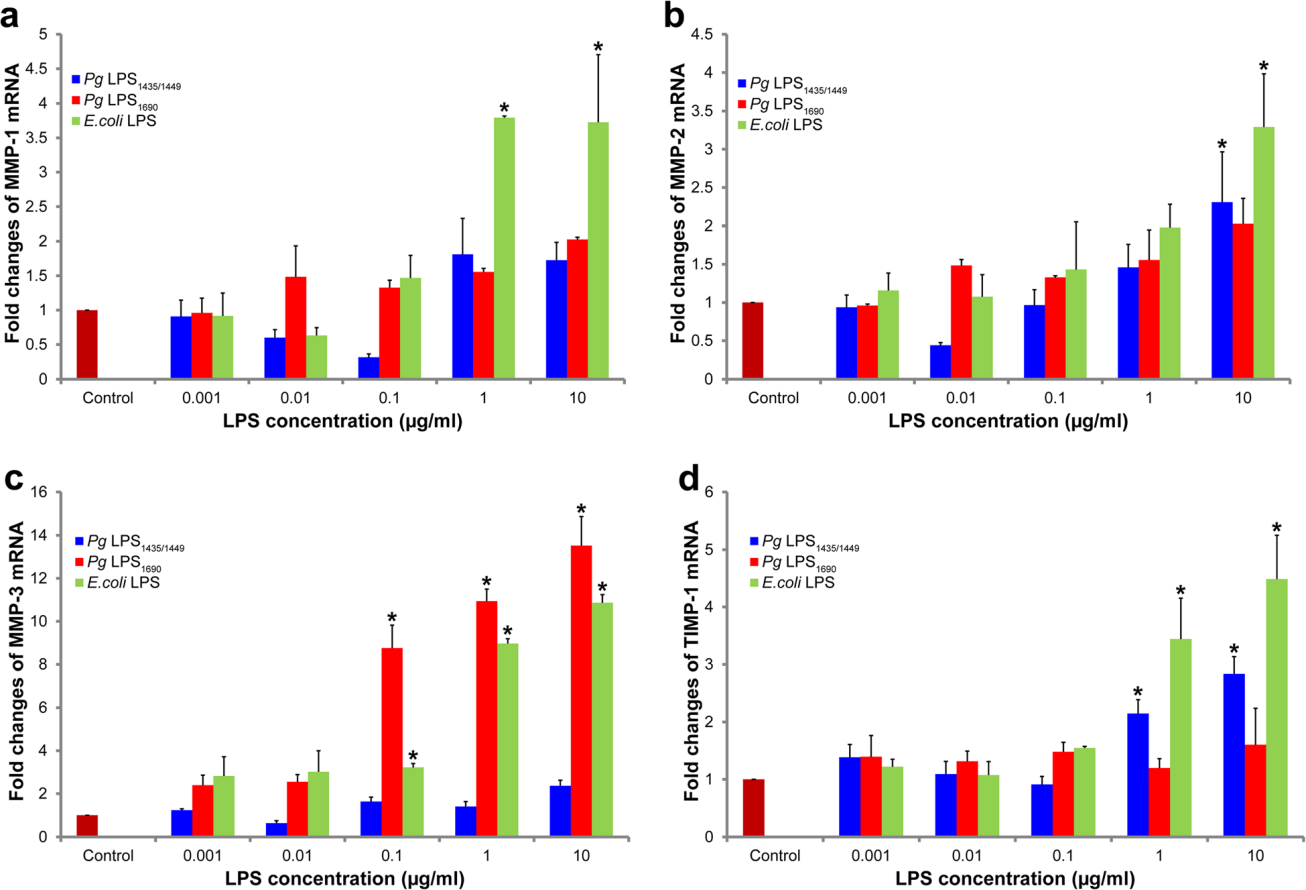
**Dose-dependent expression of MMPs 1−3 and TIMP-1 mRNAs in *P. gingivalis *LPS-treated HGFs.** Expression of MMP-1 (**a**), MMP-2 (**b**) MMP-3 (**c**) and TIMP-1(**d**) mRNAs after the stimulation of *P. gingivalis* (*Pg*) LPS_**1**435/1449_, LPS_1690_ and *E. coli* LPS in a dose-dependent assay (1 ng/ml, 10 ng/ml, 100 ng/ml, 1 μg/ml and 10 μg/ml) for 24 h. The expression of mRNAs was measured by real-time qPCR. Each bar represents the mean ± SD of three independent experiments with three replicates. *Significant difference (*p* < 0.05) as compared with the controls without LPS treatment.

The dose-dependent experiments showed that both *P. gingivalis* LPS_1435/1449_ and LPS_1690_ differentially modulated the expression of MMP-3 transcript. The latter (0.1-10 μg/ml) markedly upregulated the expression of MMP-3 mRNA while the former did not affect the expression (Figure 1c). Similarly, *E. coli* LPS (0.1-10 μg/ml) significantly upregulated MMP-3 expression. Both isoforms of *P. gingivalis* LPS upregulated to different extent the expression of MMP-1 and MMP-2 mRNAs, while *E. coli* LPS significantly upregulated the expression of these transcripts (Figures 1a and b). TIMP-1 mRNA expression was significantly induced in *P. gingivalis* LPS_1435/1449_- and *E. coli* LPS-treated cells, and no significant induction was observed following *P. gingivalis* LPS_1690_ stimulation (Figure 1d).

**Figure 2:**
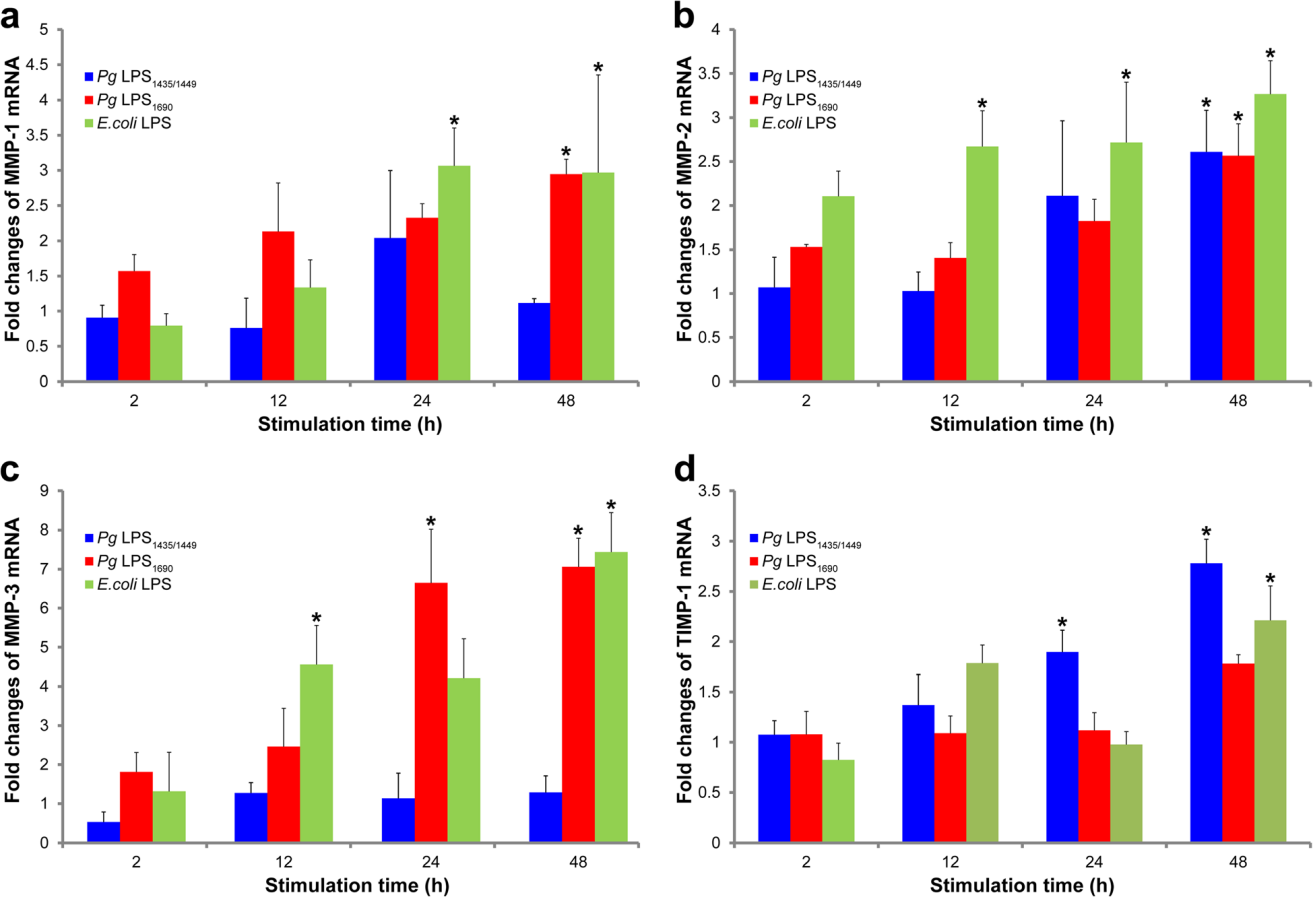
**Time-dependent expression of MMPs 1−3 and TIMP-1 mRNAs in *P. gingivalis *LPS-treated HGFs.** Expression of MMP-1 (**a**), MMP-2 (**b**) MMP-3 (**c**) and TIMP-1(**d**) mRNAs after the stimulation of *P. gingivalis* (*Pg*) LPS_**1**435/1449_ (1 μg/ml), LPS_1690_ (1 μg/ml) and *E. coli* LPS (1 μg/ml) in a time-dependent assay (2–48 h). The expression of mRNAs was measured by real-time qPCR. Each bar represents the mean ± SD of three independent experiments with three replicates. *Significant difference (*p* < 0.05) as compared with the controls without LPS treatment.

Notably, MMP-3 transcript was differentially expressed in the cells treated by the two isoforms of *P. gingivalis* LPS. *P. gingivalis* LPS_1690_ significantly upregulated MMP-3 mRNA expression at 24 and 48 h, while *E. coli* LPS showed prompt expression at 12 h (Figure 2c). MMP-2 mRNA was significantly upregulated by both *P. gingivalis* LPS_1435/1449_ and LPS_1690_ at 48 h (Figure 2b), and MMP-1 transcript was significantly upregulated by *P. gingivalis* LPS_1690_ (Figure 2a). *E. coli* LPS significantly upregulated both MMP-1 and MMP-2 mRNA expression. TIMP-1 transcript was differently modulated by *P. gingivalis* LPS_1435/1449_ and LPS_1690._ The former significantly upregulated its expression at 24 and 48 h, so did *E. coli* LPS at 48 h.

### *P. gingivalis* LPS_1690_ significantly upregulates MMP-3 protein expression

**Figure 3:**
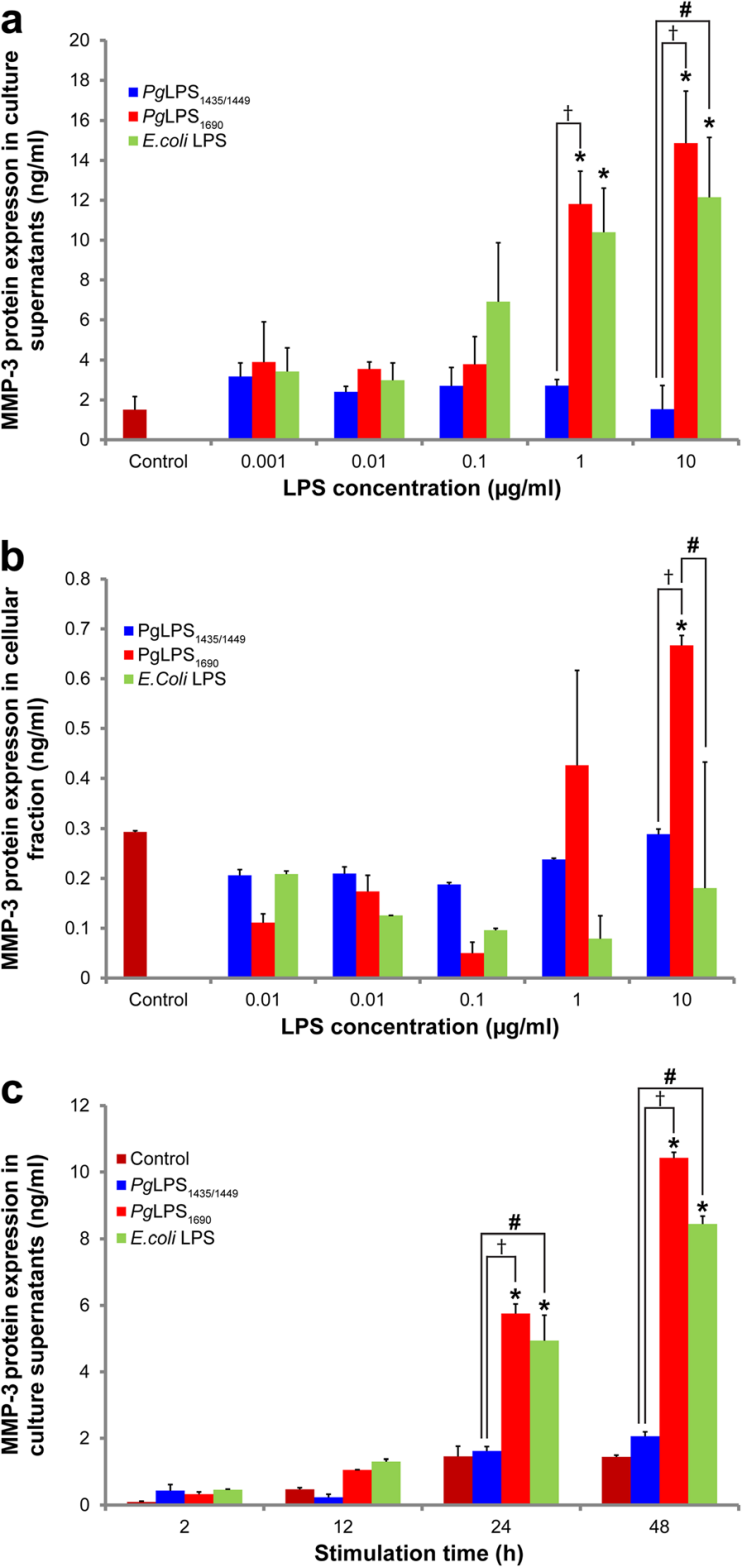
***P. gingivalis *LPS**_**1690 **_**significantly upregulates the expression of MMP-3 proteins.** Expression of MMP-3 proteins in the culture supernatants (**a**) and cellular fractions (**b**) of HGFs after the stimulation of *P. gingivalis (Pg)* LPS_1435/1449_, LPS_1690_ and *E. coli* LPS in a dose-dependent assay (1 ng/ml, 10 ng/ml, 100 ng/ml, 1 μg/ml and 10 μg/ml) for 24 h. Time-dependent expression of MMP-3 proteins in the culture supernatants (**c**) of HGFs after the stimulation of *P. gingivalis* LPS_**1**435/1449_ (1 μg/ml), LPS_1690_ (1 μg/ml) and *E. coli* LPS (1 μg/ml) for 2–48 h. The protein expression levels were measured by ELISA. Each bar represents the mean ± SD of two independent experiments with three replicates. Significant difference as compared with the controls without LPS treatment, *p < 0.05. Significant difference between the cells treated with *P. gingivalis* LPS_1435/1449_ and LPS_1690_ respectively, ^†^*p* < 0.05. Significant difference between the cells treated with *P. gingivalis* LPS and *E. coli* LPS respectively, ^#^*p* < 0.05.

Both dose- and time-dependent experiments showed that MMP-3 protein was differentially modulated by *P. gingivalis* LPS_1435/1449_ and LPS_1690_ in consistent with its transcript expression profile (Figure 3). *P. gingivalis* LPS_1690_ at 1 μg/ml and 10 μg/ml significantly upregulated MMP-3 protein expression in a time-dependent manner (12–48 h) (Figure 3c). The MMP-3 level detected in the culture supernatant was greatly higher than that in the cellular fraction (Figures 3a and b). Similar observations occurred in *E. coli* LPS-treated cells. Moreover, the MMP-3 level induced by *P. gingivalis* LPS_1690_ was significantly greater than that stimulated by *P. gingivalis* LPS_1435/1449_ (Figures 3a-c).

**Figure 4:**
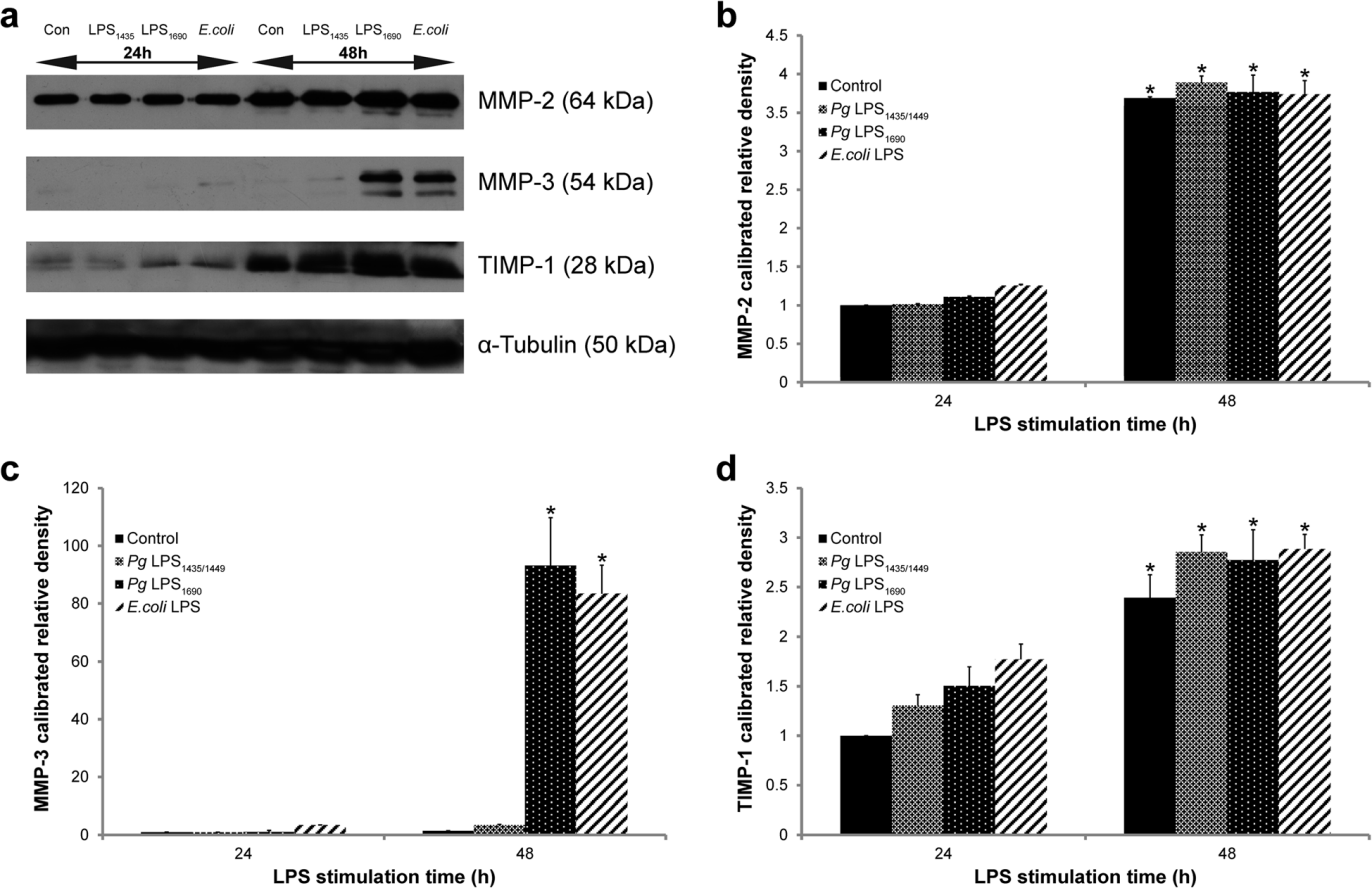
**MMP-2 and −3 as well as TIMP-1 protein expression in *P. gingivalis *LPS- and *E. coli *LPS-treated HGFs.** Confluent HGFs were stimulated with *P. gingivalis (Pg)* LPS_1435/1449_ (1 μg/ml), LPS_1690_ (1 μg/ml) and *E. coli* LPS (1 μg/ml) at 24 h and 48 h. Culture supernatants of 40 μg were subjected to SDS-PAGE and probed with anti-rabbit polyclonal MMP-2 (1:1000), MMP-3 (1:1000) and TIMP-1 (1:1000) antibodies. Blots were re-probed with α-Tubulin to confirm equal loading in samples. MMP-2: 64 kDa; MMP-3: 54 kDa; TIMP-1: 28 kDa and Tubulin: 50 kDa (**a**). Quantification of band intensities was performed by ImageJ software. The fold increase values of proteins MMP-2 (**b**), MMP-3 (**c**) and TIMP-1 (**d**) as compared with α-Tubulin are shown in the graphs. One representative blot was shown from three independent experiments. *Significant difference (*p* < 0.05) as compared with the data at 24 h.

Next, western blot analysis confirmed that MMP-3 protein markedly increased in *P. gingivalis* LPS_1690_- and *E. coli* LPS-treated cells at 48 h, while *P. gingivalis* LPS_1435/1449_ did not induce MMP-3 at a notable level (Figures 4a and c).

### The MMP-2 protein expression is not significantly affected by *P. gingivalis* LPS and *E. coli* LPS

**Figure 5:**
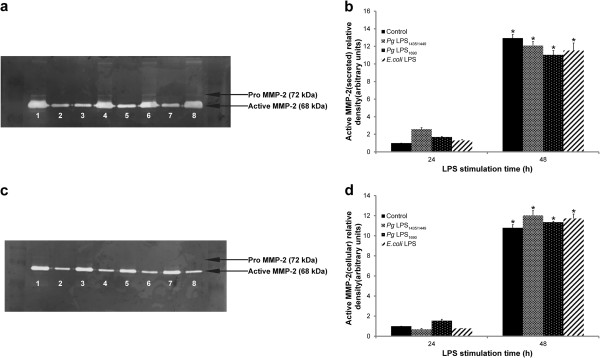
**Detection of MMP-2 in supernatant (a) and cellular fraction (c) of HGFs by gelatin zymography and molecular weight positions of pro-MMP-2 (72 kDa) and active-MMP-2 (68 kDa). **5a: Lane1: molecular weight marker; Lane 2: untreated conditioned medium at 48 h; Lane 3: untreated conditioned medium at 24 h; Lanes 4–5: *P. gingivalis (Pg)* LPS_1435/1449_ -treated culture medium at 24 h and 48 h; Lanes 6–7: P. gingivalis LPS_1690_ -treated medium at 24 h and 48 h; Lanes 8–9: E. coli LPS-treated culture medium at 24 h and 48 h, respectively. 5c: Lane1: Marker; Lanes 2–3: untreated cellular component at 48 h and 24 h; Lanes 4–5: *P. gingivalis (Pg)* LPS_1435/1449_ -treated cellular component at 48 h and 24 h; Lanes 6–7: *P.gingivalis* LPS_1690_- treated cellular component at 48 h and 24 h; Lanes 8–9: *E-coli* LPS-treated cellular component at 48 h and 24 h, respectively. Quantification of band intensities was performed by densitometry analysis using ImageJ software. The fold increase values of MMP-2 in culture supernatant (**b**) and cellular fraction (**d**) as compared with the control are shown. *Significant difference (*p* < 0.05) as compared with the data at 24 h.

Basal expression of MMP-2 was observed at 24 h, and increased at 48 h (Figures 4 and 5). With reference to the control, *P. gingivalis* LPS and *E. coli* LPS did not significantly affect the expression levels of MMP-2 proteins (Figures 4a and b). Gelatin zymograms revealed that the MMP-2 presented in two forms including pro-MMP-2 (72 kDa) and active-MMP-2 (68 kDa). In both culture supernatant (Figure 5a and b) and cellular fraction (Figure 5c and d), the activity of MMP-2 at 24 and 48 h was not significantly affected by *P. gingivalis* LPS and *E. coli* LPS.

### *P. gingivalis* LPS_1690_ induces MMP-3 expression via MAPK signaling pathway

**Figure 6:**
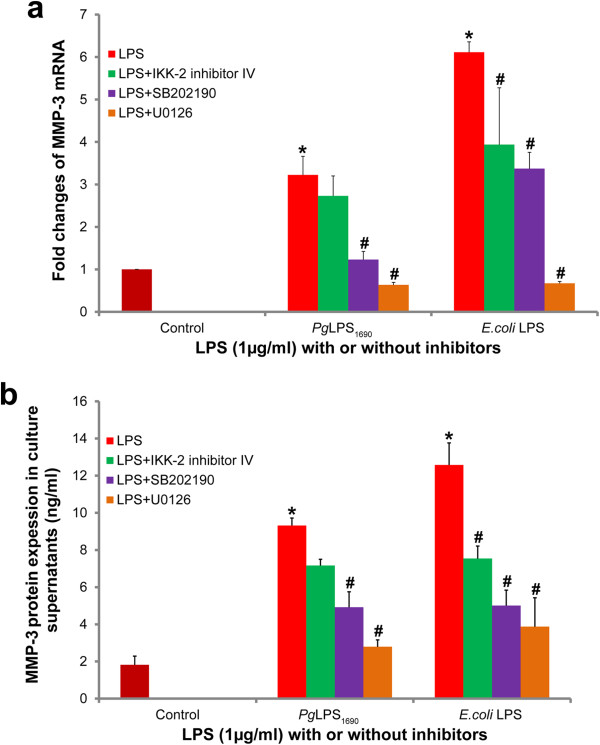
**Effects of NF-ĸB and MAPK inhibitors on *P. gingivalis *LPS**_**1690**_**-induced MMP-3 mRNA (a) and protein (b) expression in HGFs.** Cells were pretreated with IKK-2 inhibitor IV (NF-ĸB inhibitor), SB202190 (p38 MAPK inhibitor) and U1026 (ERK inhibitor) in serum free medium for 1 h, and then treated with *P. gingivalis (Pg)* LPS_1690_ (1 μg/ml) and *E. coli* LPS(1 μg/ml) for additional 12 h. Total RNA was harvested and MMP-3 mRNA levels were determined by real-time qPCR. Cell culture supernatants were collected and the protein expression level was measured by ELISA. The histogram shows quantitative representations of the MMP-3 mRNA levels of three independent experiments. Each value represents the mean ± SD. *Significant difference (*p* < 0.05) as compared with the controls. ^#^Significant difference (*p* < 0.05) as compared with the cells treated with *P. gingivalis* LPS_1690_ or *E. coli* LPS alone.

Blocking assays were performed to elucidate the involvements of NF-ĸB and MAPK signaling pathways of *P. gingivalis* LPS_1690_ induced MMP-3 expression in HGFs. Both ERK inhibitor (U1026) and p38 MAPK inhibitor (SB202190) significantly suppressed the expression levels of MMP-3 transcript (Figure 6a) and protein (Figure 6b) in *P. gingivalis* LPS_1690_- and *E. coli* LPS-treated cells. Notably, U1026 inhibited MMP-3 expression to a greater extent with reference to SB202190. The expression of MMP-3 was not significantly reduced by IKK-2 inhibitor IV in *P. gingivalis* LPS_1690_-treated cells, whereas it significantly suppressed MMP-3 in *E. coli* LPS-treated cells (Figure 6).

## Discussion

Periodontal disease is a complex inflammatory disease initiated by pathogenic plaque biofilms and results in destruction of tooth-supporting tissues and alveolar bone [17,18]. Proteolytic enzymes like MMPs play a major role in the degradation of collagens in periodontal tissues. The expression and regulation of MMPs and TIMPs in HGFs are therefore crucial for maintenance of tissue homeostasis and periodontal health. Although many studies have been performed to elucidate the mechanisms involved in the synthesis and regulation of MMPs in periodontal research, no studies are available on the effect of *P. gingivalis* LPS structural heterogeneity on the expression of MMPs and the underlying regulatory mechanisms.

MMP-3 is known as stromelysin which has both elastinolytic and collagenolytic activities that degrade basement membrane components such as laminin, elastin fibronectin as well as collagen types II, III, IV, V, IX, X and XI [8,19]. Its level could significantly increase following the stimuli of pro-inflammatory cytokines, growth factors and LPS [14,20-22]. It has been shown that HGFs could upregulate the expression of MMP-3 due to the effects of pro-inflammatory cytokines such as IL-1β and TNF-α [23-25]. The current study showed that the expression of MMP-3 mRNA and protein was markedly upregulated by *P. gingivalis* LPS_1690_, whereas no induction was observed in cells treated with *P. gingivalis* LPS_1435/1449_, indicating that the heterogeneous lipid A structures of *P. gingivalis* LPS may differentially modulate the expression of MMP-3 in HGFs. Moreover, TIMP-1 expression was differently modulated by the two isoforms of *P. gingivalis* LPS as well. It functions as an inhibitor of MMPs by forming non-covalent complexes with MMPs. It has recently been shown that MMP-3 and TIMP-1 variants may significantly contribute to chronic periodontitis and disease progression [26]. The imbalance between MMPs and TIMPs has been implicated in periodontal tissue destruction [27].

*P. gingivalis* has long been recognized as a major periodontopathogen [28]. Recently, it is regarded as a keystone pathogen due to its ability to significantly influence the oral microbial community by modulating the innate host response [29,30]. Moreover, this bacterium adopts multiple pathogenic mechanisms to evade or subvert the host immune system [31-33]. Notably, *P. gingivalis* LPS exhibits significant structural heterogeneity with both isoforms of LPS_1435/1449_ and LPS_1690_, and our recent studies show that they differentially affect the innate host defense and underlying signaling pathways, thereby contributing to the pathogenesis of periodontal disease [4,34,35]. The current observation that the different isoforms of *P. gingivalis* LPS modulate the expression of MMP-3 and TIMP-1 may represent an additional pathogenic mechanism adopted by this noxious species to disturb the physiological tissue remodeling and tissue homeostasis, leading to the initiation of periodontal disease.

*P. gingivalis* and its virulence attributes such as LPS can stimulate various cells types to secrete MMPs including MMP-3 [36,37]. On the contrary, some studies have suggested that *P. gingivalis* LPS may not induce MMPs such as MMP-1, -2 and −9 [38]. A study performed on gingival epithelial cells using *P. gingivalis* LPS and *E. coli* LPS showed that neither LPS nor IL-1β induced MMP-2 or MMP-9 [39]. Studies on tissue models such as synovial membranes dissected from rat knee joints showed induction of MMP-1, -3 and −9 mRNA levels but not MMP-2 in response to LPS stimulation [40]. However, foregoing studies have not considered the heterogeneous nature of bacterial LPS lipid A structures. Therefore, the conflicting findings of the previous studies could to some extent be due to different isoforms of *P. gingivalis* LPS as demonstrated in the present study.

In the present study, *E. coli* LPS-treated HGFs exhibited rapid and significant induction of MMPs 1 and 2 mRNAs with reference to the cells treated with *P. gingivalis* LPS_1690_. One possibility for this observation may be the higher responsiveness of HGFs to hexa-acylated nature of the *E. coli* LPS as compared to the penta-acylated structure of *P. gingivalis* LPS_1690_. This notion is consistent with previous findings that *E. coli* LPS is a potent inducer of the production of MMPs in fibroblast-like synovial cells and rat chondrocytes, as well as other innate host response molecules in HGFs and gingival/oral epithelia [41,42]. Moreover, it was noted that both *P. gingivalis* LPS_1435/1449_ and *E. coli* LPS significantly upregulated the expression of MMP-2 mRNA but not its protein as compared to the controls. A number of factors may account for this finding, such as the stability of mRNA, its processing and splicing patterns, half-life of the target protein and post-translational modifications [43,44]. Therefore, in the present study increase in MMP-2 mRNA expression level may not be necessarily reflected at its protein level.

TIMPs exhibit high affinity for binding with MMPs and lead to inhibition of their activities. In the present study, TIMP-1 mRNA was upregulated by *P. gingivalis* LPS_1435/1449_-treated HGFs, while no significant up-regulation was observed in *P. gingivalis* LPS_1690_-stimulated cells. The current results may not be comparable with previous studies in which the structural heterogeneity of LPS was not fully considered [45-49]. This omission may account for the conflicting reports in the literature. Hence, some studies have observed lower TIMP-1 levels in the conditioned media of HGFs in response to *P. gingivalis* LPS [49]. In contrast, other studies have noted the increased expression level of TIMP-1 in gingival crevicular fluid of periodontitis patients [45,47]. Moreover, periodontal treatment could alter the balance between MMP-3 and TIMP-1 [46,48]. Based upon the current findings, further study may be warranted to explore the association of different isoforms of *P. gingivalis* LPS with periodontal conditions in periodontal patients and the possible effect of periodontal treatment on the expression of these LPS isoforms by *P. gingivalis*. In addition, the discrepancy observed in TIMP-1 mRNA and protein expression following the stimulation of both *P. gingivalis* LPS_1435/1449_ and *E. coli* LPS in HGFs could be due to the complex regulation of transcription and translation [43,44].

LPS is the major immuno-stimulatory component of *P. gingivalis* which has shown to be capable of interacting with TLRs. Binding of LPS to TLRs activates the downstream signal transduction pathways such as NF-ĸB and MAPK [50,51]. Previous studies have suggested that the activation of MMPs could be through both NF-ĸB and MAPK signaling [23,52-54]. The present study demonstrated that p38 MAPK and ERK are critically involved in *P. gingivalis* LPS_1690_- and *E. coli* LPS-induced expression of MMP-3 in HGFs. This finding is supported by a previous study that p38 MAPK and ERK1/2 pathways are essential for the expression and regulation of MMPs in various cell types in response to LPS [54]. ERK, JNK and p38 MAPK pathways play vital roles in regulating the expression of MMPs induced by various stimulants such as cytokines [53,55,56]. It is noteworthy that the nature of the stimuli could result in specific signal transduction pathway in the same cell type. For instance, MAPK inhibitor significantly reduced the MMP-3 production in HGFs stimulated with IL-1β, but not with epidermal growth factor [23]. In addition, NF-ĸB pathway may be involved in regulation of MMP-3 expression in rabbit dermal fibroblasts, human saphenous vein and rabbit aortic smooth muscle cells [57,58]. The present study showed that NF-ĸB signaling is not critically involved in LPS-induced MMP-3 expression in HGFs. Notably, the MAPK pathway but not NF-κB was significantly involved in the regulation of MMP-3 expression in HGFs in both mRNA and protein levels. Previous studies have also proven that the expression of MMP-3 is mainly mediated through P38 MAPK, ERK and tyrosine kinase pathways, but not through NF-κB pathway [23,59,60]. Moreover, although a study reported that the activation of NF-κB could be important for MMP-3 secretion, no consensus NF-κB binding site was identified in the MMP-3 gene promoter [61,62]. It suggests that NF-κB may regulate the expression of this gene through different binding sites or interacting with other transcription factors [59]. Therefore, within the context and limitations of the present study, it is tempting to speculate that MAPK pathway may be crucial for MMP-3 expression in HGFs in response to *P. gingivalis* LPS_1690_. Furthermore, it would be interesting to extend the study to other cells types in human gingiva like gingival epithelial cells to ascertain whether MAPK pathway plays a predominant role in the expression and regulation of MMP-3 in other cells of oral tissues.

## Conclusions

The present study reveals that HGFs significantly express MMP-3 in response to penta-acylated *P. gingivalis* LPS_1690_ and hexa-acylated *E. coli* LPS, but not to the tetra-acylated *P. gingivalis* LPS_1435/1449_ in HGFs. Blocking p38 MAPK and ERK pathways significantly down-regulates *P. gingivalis* LPS_1690_- and *E. coli* LPS-induced expression of MMP-3. These findings indicate that the heterogeneous lipid A structures of *P. gingivalis* LPS differentially modulate the expression of MMP-3 in HGFs, which may play a role in periodontal pathogenesis.

## Methods

### Preparation, purification and identification of *P. gingivalis* LPS

*P. gingivalis* LPS was isolated from *P. gingivalis* ATCC 33277 (the American Type Culture Collection, Rockville, MD). LPS was prepared by the cold MgCl_2_-Ethanol procedure followed by lipid extraction and conversion to sodium salts as previously described [63,64]. Optical densities were measured at 280 nm and 260 nm to verify the nucleic acid and protein contamination. LPS preparations were further treated to remove the endotoxin protein and the final protein contamination was less than 0.1% [65]. The fatty acid composition of *P. gingivalis* LPS was further analysed by Gas chromatographic-mass spectroscopy. Then two separate extractions of *P. gingivalis* LPS with tetra- (LPS_1435/1449_) and penta-acylated (LPS_1690_) lipid A structures were generated, and their structures were verified by matrix-assisted laser desorption ionization time-of-flight mass spectrometry. The canonical hexa-acylated LPS of *Escherichia coli* JM 83-wild type strain was used as the reference [66].

### Cell culture

HGFs were obtained from Sciencell research laboratories (Carlsbad, CA, USA) and cultured according to the manufacturer’s instructions [67,68]. Continuous subcultures up to 10th passage contained homogeneous, slim and spindle-shaped cells growing in characteristic swirls. Third to fourth passages of HGFs without any signs of senescence were used for all experiments as described in our previous study [4].

### Stimulation of HGFs by heterogeneous *P. gingivalis* LPS

The cells suspended at 10^5^ cell/ml were seeded on six-well-plates and grown until confluent at 37°C with 5% CO_2_ in a culture medium for fibroblasts consisting of basal medium with 2% fetal bovine serum, penicillin/streptomycin (0.01% w/v) and fibroblast growth supplement. Once the cells were over 90% confluent, fibroblast medium (FM) was replaced entirely with serum free and animal component free-medium (FM-acf) for the dose- and time-dependent experiments. In the dose-dependent assay, cells were stimulated with *P. gingivalis* LPS_1435/1449_, *P. gingivalis* LPS_1690_ or *E. coli* LPS in the media containing various doses of LPS (0.001 μg/ml −10 μg/ml). Subsequently, 1 μg of LPS was selected as the appropriate dose for the following time-dependent experiments. Cells were incubated with *P. gingivalis* LPS or *E. coli* LPS at 1 μg/ml and harvested at 2, 12, 24 and 48 h. Cells without LPS treatment were designated as the controls. Culture supernatants were collected and centrifuged to remove the cellular debris and stored at −70°C for subsequent protein assays. Cellular fraction was then washed with PBS and collected for mRNA and protein extraction.

### RNA extraction, cDNA synthesis and real-time qPCR

Total RNA extraction, cDNA transcription and real-time qPCR for MMPs1-3 and TIMP-1 were performed as previously described [17]. In brief, total RNA was extracted from the homogenized HGFs using RNeasy Mini Kit (Qiagen, Hilden, Germany) according to the manufacturer’s instructions [35]. cDNA was synthesized by reverse transcriptase-PCR at 43°C for 90 min in a 20 μl of reaction mixture containing 1 μg of total RNA, 1 μl (200 U) of SuperScript™ First-Strand Synthesis System (Invitrogen Corp., Carlsbad, CA, USA), 0.5 μg of oligo dT-primer, first-strand buffer, 10 mM DTT, and 1 mM dNTPs. A control reaction was performed without reverse transcriptase for all samples to verify the absence of genomic DNA contamination. Real-time qPCR was then performed by using the StepOne Real-Time PCR System (Applied Biosystems, Foster City, CA) in at least three separate experiments. Amplification reactions were undertaken in 20 μl of reaction mixture containing 10 μl of Power SYBR® Green PCR Master Mix, 1 μl of cDNA template and 1 μl of each pair of primers for the targeting cytokine genes (Sigma, St. Louis, MO, USA). Real-time primer pairs were designed using ABI software to amplify a sequence that contains two or more exons whenever possible. The amplification efficiencies of the primers used were above 90%. The specific sequences for each pair of primers are listed in Table 1. β-actin was amplified as an internal control. The real-time qPCR reaction conditions were set at 95°C for 10 min followed by 40 cycles at 95°C for 15 s and 60°C for 60 s. The results were analyzed using the comparative cycle threshold (Ct) method as previously described [35]. The expression level of each gene was normalized to a β-actin (ΔCt) and the fold changes for each gene were calculated by comparing the test and control samples from the ΔΔCt values.

**Table 1:** Nucleotide sequence of real-time qPCR primers

**Primers**	**Sequence (5**^**′**^**-3**^**′**^**)**
MMP1-F	ATG CTG AAA CCC TGA AGG TG
MMP1-R	CTG CTT GAC CCT CAG AGA CC
MMP2-F	AGG GCA CAT CCT ATG ACA GC
MMP2-R	ATT TGT TGC CCA GGA AAG TG
MMP3-F	GCA GTT TGC TCA GCC TAT CC
MMP3-R	GAG TGT CGG AGT CCA GCT TTC
TIMP1-F	CTG TTG TTG CTG TGG CTG AT
TIMP1-R	TCC GTC CAC AAG CAA TGA GT
β-actin -F	TTG GCA ATG AGC GGT T
β-actin -R	AGTTGAAGGTAGTTTCGTGGAT

### Total protein extraction and detection of MMP-3 by ELISA

Total proteins were extracted from homogenized HGFs using CellLytic^TM^ MT-mammalian cell lysis extraction reagent (Sigma, USA). Protein concentrations in both of the cell-bound fraction and culture supernatant were measured respectively by BCA protein assay kit (Pierce, Thermo Scientific, USA) according to the manufacturer’s instructions. Enzyme-linked immunosorbent assay (ELISA) was performed to confirm the expression of MMP-3 proteins (BioRad Laboratories, Hercules, CA, USA). The protein expression in both cell lysate and culture supernatants were measured following manufacturer’s instruction with the minimal detectable concentration of 0.009 ng/ml. No cross reactivity or no interference was observed with recombinant MMP-3. The absorbance values were determined by a micro-plate reader (Victor, Vienna, VA, USA) at optical absorbance of 450 nm and the final concentration was determined with reference to a standard curve. Experiments were repeated two times with three biological replicates.

### Western blot analysis for MMP-2, -3 and TIMP-1 proteins

Total cell lysates were prepared and 40 μg of cellular extracts were separated by 10% SDS-PAGE gel and subsequently transferred onto a polyvinylidene difluoride membrane (PVDF). The proteins were then blocked against the protein-free blocking buffer (Pierce, Thermo Scientific) for 1 h. Afterwards, membranes were incubated overnight at 4°C with primary antibodies against polyclonal rabbit anti-human IgG; MMP-2 (1:1000; Cell signaling), MMP-3 (1:1000; BioVendor) and TIMP-1 (1:1000; Cell signaling), and incubated with horseradish peroxidase (HRP) conjugated anti-rabbit secondary antibodies (1:10000). Visualization of the immunoreactive proteins were accomplished by the use of SuperSignal West Pico Chemiluminescent Substrate (Thermo Scientific, Rockford, IL, USA) and exposed to X-ray films. α-Tubulin was used as the internal loading control (1:1000; Cell signaling). The detected bands were scanned on a calibrated densitometer, GS-800 and assessed by the imageJ software-based analysis (http://rsb.info.nih.gov/ij/) to quantify the integrated density.

### Gelatin zymography for enzymatic activity of MMP-2

SDS-PAGE gelatin zymography was performed to observe the enzymatic activity of MMP-2. Supernatants and cellular proteins were collected from cells grown in serum-free medium at 24 h and 48 h as described above. Centrifugal filter devices (Amicon Ultra-0.5-Millipore USA) with a cut off value of 30000 NMWL (Nominal Molecular Weight Limit) were used to concentrate the supernatants. Culture supernatants or cellular extracts (40 μg) were mixed with 2 × non-reducing sample buffer without β-mercaptoethanol (0.125 M Tris–HCl at pH 6.8, 4% SDS, 20% glycerol and 0.05% bromophenol blue). Proteins were separated by 10% Tris-glycine polyacrylamide gel copolymerized with 0.1% gelatin as a substrate. After electrophoresis, gels were washed in renaturation buffer (2.5% Triton X-100 in 50 mM Tris–HCl at pH 7.5) for 1 h and incubated for 20 h at 37°C in incubation buffer (0.15 M NaCl, 10 mM CaCl2 and 0.02% NaN3 in 50 mM Tris–HCl at pH 7.5). Gels were stained with 5% Coomassie blue and destained with 7% methanol and 5% acetic acid to reveal zones of lysis within the gelatin matrix. Areas of enzymatic activity appeared as clear bands over the dark background.

### Signal transduction pathways involved in LPS-induced MMP-3 expression in HGFs

Specific pharmacological inhibitors for NF-κB activity, IKK-β inhibitor (IKK-2 inhibitor IV), p38 MAPK activity (SB202190) and ERK activity (U1026) were used to investigate two major signaling pathways potentially involved in the expression and regulation of MMP-3 in HGFs in response to heterogeneous *P. gingivalis* LPS. Each inhibitor was first dissolved in dimethyl sulfoxide (DMSO) and diluted in DPBS. Cells were pretreated with kinase inhibitors, including 10 μmol/L of IKK-2 inhibitor IV (Merck, USA), 10 μmol/L of SB202190 (Calbiochem Biosciences Inc, La Jolla, CA, USA) and 15 μmol/L of U1026 (Cell Signaling, USA) respectively for one hour, prior to stimulation with LPS. Afterwards, 1 μg/ml of LPS was added to the medium and cells were incubated for another 12 h. Culture supernatants were collected for analyzing the MMP-3 expression by ELISA. Extracted RNA was subjected to real-time qPCR to detect the MMP-3 transcript expression. Positive controls were the supernatants from the cells treated with LPS alone, whereas the negative controls were incubated with the culture medium alone. In addition, the cells treated with DMSO alone were considered as the vehicle control (data not shown).

### Statistical analysis

All experiments were repeated in three assays for real-time qPCR and two assays for ELISA. Results of the experiments were presented as the mean ± SD. The statistical significance of difference between the data sets from the dose-dependent assay was evaluated by student t-test, one-way analysis of variance (ANOVA) and post hoc testing with Bonferroni and LSD methods. Additionally, the repeated-measures of ANOVA were used to determine the differences between data sets from the time-dependent assay. A *p-*value < 0.05 was considered statistically significant. All statistical analysis was performed using a software program (SPSS 19.0, SPSS Inc, Chicago, IL, USA).

## Abbreviations

Pg: *P. gingivalis*; LPS: Lipopolysaccharides; HGFs: Human gingival fibroblasts; MMPs: Matrix metalloproteinases; TIMP: Tissue inhibitors of metalloproteinases; qPCR: Quantitative PCR; ELISA: Enzyme linked immunosorbent assay; NF-κB: Nuclear factor-kappaB; MAPK: Mitogen-activated protein kinase.

## Competing interests

The authors declare that they have no competing interests.

## Authors’ contributions

TDKH, CJS and LJJ conceived the study. RPD carried out the preparation, purification and identification of *P. gingivalis* LPS. TDKH and CJS performed the cell culture of HGFs, RNA extraction, cDNA synthesis and real-time qPCR, ELISA, Western blot, gelatin zymography, and detection of signal transduction pathways. RPD, CYW, YW and LJJ were involved in supervision of the experiments and provided reagents and materials. TDKH, CJS and LJJ analyzed the data, and wrote the manuscript. All authors read and approved the final manuscript.
